# Efficient *de novo* assembly of large and complex genomes by massively parallel sequencing of Fosmid pools

**DOI:** 10.1186/1471-2164-15-439

**Published:** 2014-06-06

**Authors:** Andrey Alexeyenko, Björn Nystedt, Francesco Vezzi, Ellen Sherwood, Rosa Ye, Bjarne Knudsen, Martin Simonsen, Benjamin Turner, Pieter de Jong, Cheng-Cang Wu, Joakim Lundeberg

**Affiliations:** School of Biotechnology, Science for Life Laboratory, KTH Royal Institute of Technology, Box 1031, 171 21 Solna, Sweden; Department of Biochemistry and Biophysics, Science for Life Laboratory, Stockholm University, Box 1031, 171 21 Solna, Sweden; Lucigen Corporation, 2120 W Greenview Dr., Suite 9, Middleton, WI 53562 USA; CLC bio A/S, Silkeborgvej 2, 8000 Aarhus C, Denmark; BACPAC Resources, Children’s Hospital of Oakland Research Institute, Bruce Lyon Memorial Research Building, Oakland, California 94609 USA; Department of Microbiology, Bioinformatics Infrastructure for Life Sciences, Science for Life Laboratory, Tumour and Cell Biology, Karolinska Institutet, 17177 Stockholm, Sweden; Department of Cell and Molecular Biology, SciLifeLab, WABI, Uppsala University, Uppsala, Sweden; Intact Genomics Inc, 1100 Corporate Square Drive, Suite 257, St. Louis, Missouri USA

## Abstract

**Background:**

Sampling genomes with Fosmid vectors and sequencing of pooled Fosmid libraries on the Illumina platform for massive parallel sequencing is a novel and promising approach to optimizing the trade-off between sequencing costs and assembly quality.

**Results:**

In order to sequence the genome of Norway spruce, which is of great size and complexity, we developed and applied a new technology based on the massive production, sequencing, and assembly of Fosmid pools (FP). The spruce chromosomes were sampled with ~40,000 bp Fosmid inserts to obtain around two-fold genome coverage, in parallel with traditional whole genome shotgun sequencing (WGS) of haploid and diploid genomes. Compared to the WGS results, the contiguity and quality of the FP assemblies were high, and they allowed us to fill WGS gaps resulting from repeats, low coverage, and allelic differences. The FP contig sets were further merged with WGS data using a novel software package GAM-NGS.

**Conclusions:**

By exploiting FP technology, the first published assembly of a conifer genome was sequenced entirely with massively parallel sequencing. Here we provide a comprehensive report on the different features of the approach and the optimization of the process.

We have made public the input data (FASTQ format) for the set of pools used in this study:

ftp://congenie.org/congenie/Nystedt_2013/Assembly/ProcessedData/FosmidPools/.

(alternatively accessible via http://congenie.org/downloads).

The software used for running the assembly process is available at http://research.scilifelab.se/andrej_alexeyenko/downloads/fpools/.

**Electronic supplementary material:**

The online version of this article (doi:10.1186/1471-2164-15-439) contains supplementary material, which is available to authorized users.

## Background

While drastically increasing performance, massively parallel sequencing (next generation) technologies that use short reads have created new challenges in carrying out downstream bioinformatics analyses. One of these challenges is that complexity of the assembly task is much higher as a result of very short read lengths. The *de novo* assembly of large genomes faces particular challenges in repeat-rich regions because of higher numbers of repeat copies. Initially, helping the assembly by increasing read coverage appeared to be the most obvious remedy. However, for assembling genomic regions with redundant genome fragments the additional costs of memory, time, and price turned out to be disproportionate to the minor improvements achieved by increased sequencing depth. It is usually impossible to resolve repeats longer then the read length.

One possible solution to this problem is to use FPs. Fosmid technologies [1, 2] employ the origin of replication of the F-plasmid and the partitioning mechanisms of the *E. coli* genome to clone large (20-90 Kbp) chromosome fragments. Bacteriophage lambda packaging restricts the fragment length to ~40 Kbp. Fosmid ends can produce mate-pair (jump) libraries that facilitate the assembly of shotgun genome sequences in the absence of large-scale bacterial cloning [3, 4]. Another application of Fosmids is in obtaining material for genome-scale sequencing via a massive Fosmid-based approach in which the inserts are completely sequenced. In order to load next generation sequencers (Illumina) with required amount of DNA, hundreds or thousands of Fosmids should be combined. The FP approach enables the complexity of downstream bioinformatics analyses to be reduced in a number of ways:

1. each sampled genomic fragment is haploid within a Fosmid [5, 6] – hence assembly of the fragment is not hindered by allelic differences;
2. in repeat-rich genomes, repeats are the major reason for breaks in assembly contiguity, and the repeat assembly problem is heavily reduced when, as exemplified by assembly of the Norway spruce (*Picea abies*) genome [7], each pool of 1,000 Fosmids contained in total ~40 Mbp genomic regions compared to the challenge to assembly all WGS reads from the entire 20 Gbp genome;
3. it is not necessary to use large-memory computers in order to solve the assembly problem (whereas up to 1 TB of memory is needed to assemble a WGS read set); and
4. for certain assembly algorithms, it is important to know the approximate number of genomic fragments sampled in each instance and length limits of each region.

Using sets of contigs originating from FPs also introduces constraints that are helpful for further scaffolding of these contigs and merging them with contigs obtained from WGS. One such constraint is that any pair of contigs from the same pool is unlikely to represent the same genomic region (the probability of having at least one such overlap in a pool of 1000 Fosmids is of an order of 10^-3^), while it is more likely for contigs from different pools. Sequence similarity of two contigs from the same pool can therefore, with highest likelihood, be attributed to paralogy rather than to an allelic difference.

These advantages have led to the growing use of FP technologies in those large genome sequencing projects that do not use Sanger sequencing and BACs [8]. The FP approach was indispensable in the sequencing and assembly [7] of the genome of Norway spruce, *P. abies.* It helped to assemble many more repeat regions than it would be possible to do using WGS alone, given the large size (~20 Gbp) and complexity (70% repeat-like structures) of the genome.

In the course of the spruce sequencing project we produced and compared contigs from test FPs of different sizes, contigs from hundreds of FPs generated in a massive production process, and WGS-generated contigs. This enabled us to carry out a multilateral comparative analysis of both the technical parameters used and the biological content of the assemblies, and we present the results in this article. We also describe the FP-specific preparation of libraries, report on the optimization of the assembly process, and present features of the assembled fragments of the spruce genome.

## Methods

### Preparation of high molecular weight (HMW) genomic DNA and ~40 kb inserts

Fresh needles from shoots over-wintered since the previous year of the reference tree [7] were collected near Umeå, Sweden during late spring (25^th^ of March, 2010) and immediately frozen at -80°C.

We prepared HMW spruce genomic DNA using a modified nuclei-agarose DNA plug purification protocol. About 50 g of the frozen young spruce needles were briefly ground to a fine powder in liquid nitrogen with a mortar and pestle. The powder was gently re-suspended in 500 ml of ice cold nuclei preparation buffer (10 mM Tris, 80 mM KCl, 10 mM EDTA, 1 mM spermidine, 1 mM spermine, pH 9.4-9.5 with 0.15% β-mercaptoethanol and 0.5% Triton X-l00) and incubated on ice for 20 minutes to lyse the cells. The extract was filtered through two layers of cheesecloth and one layer of Miracloth and the nuclei were pelleted by centrifugation at 1,800 g at 4°C for 20 min. After washing the nuclei extensively with the nuclei preparation buffer (in order to minimize contamination by chloroplasts and mitochondria, five or more washes were carried out until no green color remained in the supernatant), we made 100 ~ 150 plugs by embedding the nuclei in 1% low-melting-point agarose. Each set of 50 plugs was lysed in 40 ml of lysis buffer (0.5 M EDTA; pH 9.0-9.3, 1% sodium lauryl sarcosine, and 0.1 - 0.5 mg/ml proteinase K) at 50°C for 48 hours with gentle shaking. After washing the DNA plugs, the genomic DNA was electro-eluted (100 V for 1 hour at 4°C) into a dialysis tube with 1X TAE buffer, and the HMW genomic DNA was finally extracted with phenol/chloroform/isoamyl alcohol, precipitated with isopropanol, and resuspended in TE buffer. We routinely obtained at least 200 μg of high quality cloning-ready HMW genomic DNA from ~50 grams of frozen young spruce needles.

Each 10 μg of HMW genomic DNA was sheared using a HydroShear apparatus (Digilab) with the large pore orifice at a speed code of 25. The sheared large DNA fragments were end-repaired with a DNA terminator kit (Lucigen) for 30 min at room temperature followed by heat inactivation at 70°C for 15 min. The end-repaired large fragment DNA was fractionated on a CHEF-Mapper system (BioRad) in 1% PFGE agarose, 0.5X TBE, using the following conditions: 6 V/cm, 0.1 - 2 s time ramp, 11 hrs, 120° angle, and 14°C. After excising gel slices from the unstained portion of the gel corresponding to the region between the 30 kb and 50 kb size markers, the DNA was recovered by electroelution, precipitated with isopropanol and resuspended in TE buffer at a concentration of 250 ng/μl.

### Fosmid library construction and pooling

We constructed all Fosmid libraries according to the Fosmid Cloning Kit manual (Lucigen). For one Fosmid cloning reaction per library, 10 μl of ligation mixture contained 250 ng of insert DNA, 500 ng of linearized and dephosphorylated pSMART-FOS or pNGS-FOS vector (Lucigen), 1× T4 DNA ligase buffer, and 5 units of T4 DNA ligase (Lucigen); it was incubated at room temperature for 3 hours, and the ligation reactions were then heat-inactivated at 70°C for 15 min. For Fosmid packaging, each 10 μl ligation mixture was packaged, using MaxPlax lambda packaging extracts (Epicentre), by two successive additions of 25 μl packaging extract followed by 90 min incubations at 30°C; 1 ml of phage dilution buffer (SM buffer with 0.01% gelatin) and 30 μl chloroform were then added to each packaging reaction and mixed well, and the Fosmid phage particles were stored at 4°C. For Fosmid transfection and titering, each 10 μl aliquot of phage particles was added separately, either without or with dilution, to 100 μl of prepared Replicator FOS Cells (Lucigen), and shaken at 225 rpm for 20 minutes at 37°C; the transfected Replicator FOS Cells were then plated on YT media plates containing chloramphenicol (12.5 μg/ml), X-Gal (40 μg/ml), IPTG (0.4 mM), and sucrose (5%, w/v), and incubated overnight at 37°C, after which the packaged phage titer was calculated. All Fosmid libraries were titered and stored at -80°C.

All the Fosmid pooling was carried out using the same protocol. After testing the efficacy of Illumina sequencing and assembling from pool sizes with ~100, 500, 1,000, 2,500 and 5,000 Fosmid clones, we set the size of each of the remaining 1,000 FPs at 1,000 clones. We plated each set of Fosmids from titered libraries on a Q-tray (22 cm x22cm) with 12.5 μg/ml chloramphenicol and 5% (w/v) sucrose, and incubated the trays for 24 hours at 37°C. After confirming the number of clones, the Fosmid cells from each Q-tray were collected as a pool and FP DNA was purified according to an improved alkaline lysis method. The modification consisted of preparing alkaline solution III at pH 4.8 instead of the standard value 5.5, and the mixture was processed extremely gently and carefully after addition of alkaline solution II. Finally, each FP DNA was dissolved in 300 μl of TE at a concentration of ~20 ng/μl and stored at -80°C.

### Cost of fosmid pool production

For the pools of 1000 Fosmids, we estimated the cost of consumables for production (before sequencing) as around 5 USD per one pool.

### Sequencing

All the FP samples were sequenced on an Illumina HiSeq 2000 platform as separate 300 bp insert libraries with paired end reads of 2×100 bp, using standard Illumina protocols and kits. Six tagged FPs were loaded per lane. The 650 bp paired-end libraries and the jumping libraries of insert size 2.4 Kb (both were used for scaffolding) were constructed from nuclear whole-genome shotgun DNA. To the 2.4 kbp libraries, we applied a circularization based on the protocol for the 454 platform. Its major feature was broad range of insert size and very low fraction of PE reads. Details of all these steps are described in Additional file 1: 1 and 7 to (8).

### Assembly

FPs were assembled using the CLCbio *de novo* assembler v. 4.0.6 beta for the 64-bit Linux platform (CLC bio, Aarhus, Denmark) by recruiting single cores on 8-core machines with 24 GB RAM from the Linux cluster UPPMAX (Uppsala, Sweden).

## Results

### Optimization of the FP strategy

**Figure 1:**
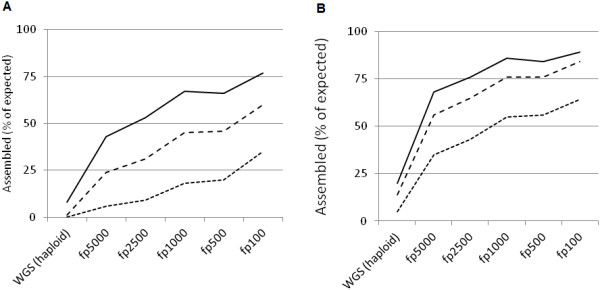
**Assembly length achieved as a function of minimal contig size (bp) and pool complexity (number of Fosmids per pool). A**: FP assembly alone. **B**: assembly with scaffolding. The leftmost point on each x-axis indicates the values achieved by haploid whole genome shotgun (WGS) sequencing. Solid, dashed, dotted lines: data for contigs longer than 5, 10, and 20 Kbp, respectively. Read coverage after read quality control and filtering was around 38x for the haploid WGS, and then 65x, 115x, 150x, 160, and 200x for the pools from fp5000 to fp100, respectively. The libraries used for scaffolding, 650 bp paired end and 2.4 kbp mate pairs, provided coverage around 20x and 25x, respectively.

In genomes with a high prevalence of repeats, like that in the spruce, one would expect repeats to be the major reason for assembly termination. By representing only a small fraction of the genome in each pool, FPs can considerably reduce the repeat problem. Ideally, one would sequence and assemble each Fosmid individually (pool size n = 1). However, the technical challenges inherent in producing hundreds of thousands of tagged sequencing libraries currently make this an impractical solution for most sequencing facilities. Achieving the best trade-off between the time and cost needed for library preparation on the one hand, and the quality of assembly results on the other, is therefore a key issue when deploying FP sequencing.To optimize the FP strategy, we produced 5 pilot pools of varying sizes (n = 100, n = 500, n = 1000, n = 2500 and n = 5000 Fosmids per pool). Each pool was assembled individually, and the assemblies resulting from the pools were compared to a 50X whole genome shotgun assembly from the same spruce individual. We measured the efficiency of assembly as total length of contigs longer than a certain threshold (5 Kbp, 10 Kbp, 20 Kbp; Figure 1) and normalized the result by dividing it by the expected total length of genomic fragments in the pool. As anticipated, the assembly metrics improved dramatically with smaller pool sizes. As it follows from Figure 1B, further improvement was achieved with scaffolding the contigs using mate pair libraries.

Another important parameter was the required depth of sequencing per pool. The FP assemblies presented at Figure 1 were performed at coverage much higher (1.7…7.9 fold) than that for the WGS assembly (38×). In order to investigate this factor, we simulated lower coverage with random sub-sampling of reads at different levels (25X, 50X, and 75X) and compared these options to the coverage actually achieved (90X-160X depending on pool) in seven example test pools (Additional file 1: Figure S1). Although assembly efficacy decreased at lower sequencing depth, even at 25X it was possible to outperform the WGS approach. The difference between 75X and full coverage was marginal. We also noted that decreasing the depth of read coverage has a greater effect on the output of longer contigs than on that of shorter ones.

The optimal pool size depends on the current costs and the level of automation available for library production at each sequencing facility. In our case, we concluded that sequencing 1000 Fosmids per pool at a target coverage depth of 75× offered the optimum trade-off between cost and quality,. In the following sections, we describe the FP production mode used for the spruce genome project, which has so far reached about 2X genomic coverage using FPs (i.e. around 1000 pools have sampled the 20 Gb genome two-fold).

### Properties of FPs and contigs

**Figure 2:**
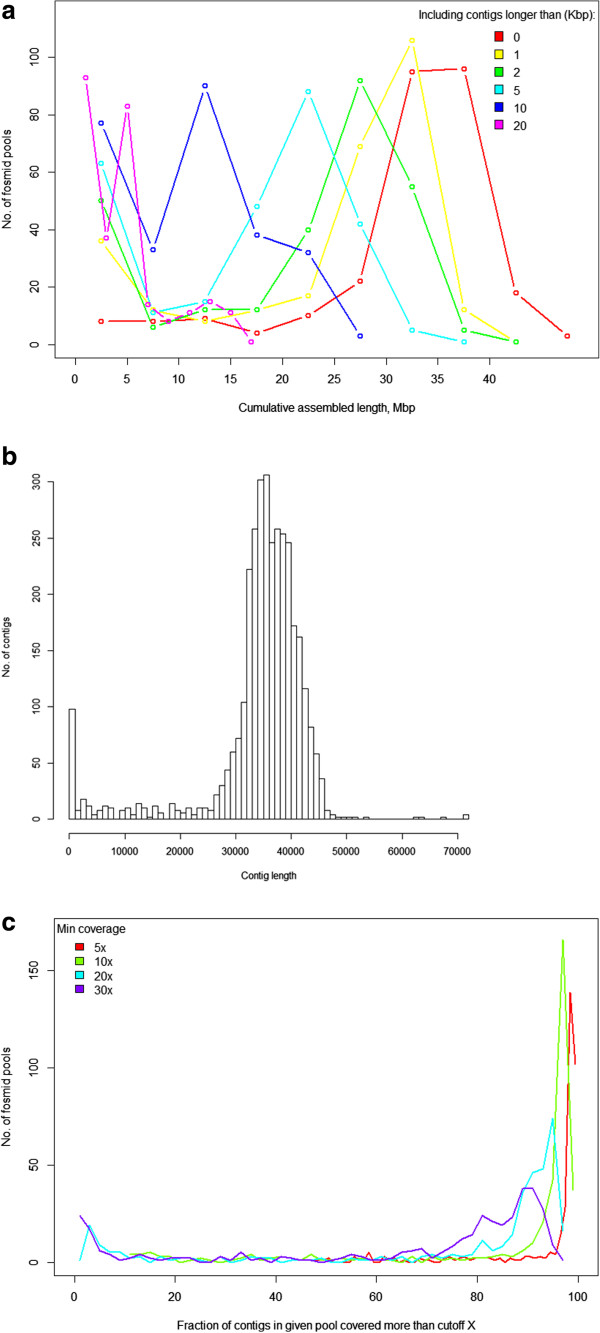
**Quality parameters of FPs. A**, cumulative assembly length of contigs from 360 FPs in the production set longer than a given threshold. Each colored line represents a distribution of total assembled length per pool by taking into account contigs longer than the specified length (0…20 kbp). **B**, length distribution of contigs that contained both fragments that matched start and end of the vector sequence (from 79 FPs produced with four different vectors). The expected range length was 27…47 Kbp, hence the outliers emerged due to either damaged Fosmids or mis-assembly. **C**, distribution of coverage across different contigs in pools (from 360 FPs in the production set). Bins of the histogram represent the number of FPs that have coverage not worse than the threshold specified in the color legend. For example, the highest point of the green line tells that there were 170 FPs in which 97% of contigs were covered at least 10x (as average along the contig length). **A**, **B**, **C**.

After sampling with vector sequences, the flanks of the latter were merged with the sampled genome fragment and could generate chimeric sequencing reads, after which contig ends could contain small fragments of the vector. This could help in evaluating length distribution of the sampled fragments and correctness of their assembly. In order to do that, we randomly selected 79 FPs which represented the four different vectors that we tested (pFosDT3, pFosDT5, pSMART-BAC_FOS, and pNGS-FOS, of which the latter was used for the massive production). We searched for vector ends in all of the 1,071,614 contigs assembled after sequencing of these pools. There were 83,855 (7.8%) contigs that contained at least one vector end. By allowing not more than 10 of the last contig base pairs to fall outside the BLAST hit to the vector end sequence, 99.8% of such vector fragments were found at the very end of the contig (the median fragment length was 46 bp). Of the 83,855 contigs investigated, 1,694 contained two vector fragments. In 1,516 (89.5%) of these cases, both vector start and vector end were found in the correct orientation. Nearly all of these “proper” contigs had lengths in the expected range (27 Kbp and greater) (Figure 2B). We utilized the number of vector/insert junctions in raw reads as a control to estimate the actual number of Fosmid clones in each pool. In general, there was good agreement between the estimates and the intended target number of Fosmids (65%…150%). Although at the earlier stage of development, it helped to understand that in some cases the true numbers of Fosmids were as low as 50% of what was aimed for.

### Contamination screening and assembly optimization

An unavoidable drawback of bacterial cloning was the presence of the Fosmid cloning vector and of hitch-hiking genomic *E. coli* DNA in the DNA preparations. As the vector length could be up to 8 Kbp, sequencing these fragments imposed an extra cost to the around 40 Kbp of the Fosmid inserts. Additionally, the sequences of the chloroplast and mitochondrial genomes of *P. abies*[7] were recurrently sampled in parallel with the nuclear DNA, hence were desirable to filter out efficiently. To achieve this, we applied two rounds of filtering against these known contaminants. Regarding *E.coli*, we considered both the whole genome sequence and, in particular, known transposable elements. Firstly, all reads were mapped to the contaminants using samtools [9]. Secondly, since the mapping might not give perfect results, assembled contaminant contigs were also identified by BLAST [10]. Both contaminant reads and contigs identified as being of non-chromosomal origin were removed before collecting statistics for assembly optimization. The fractions of DNA from vector (always under 10%, and 5.2% in the production pools) and chloroplast (always under 1%, and 0.02%in the production pools) were consistent and at the expected levels across all pools. In contrast, we noted that the levels of *E. coli* and mitochondrial DNA were very variable across pools in the early phase of the production mode, sometimes being responsible for up to 50% of the data. High levels of *E. coli* reads presumably resulted from the preparation of Fosmid DNA from *E. coli* cells, while high levels of mitochondrial DNA presumably stemmed from enrichment for mitochondrial DNA during preparation of genomic DNA from the original spruce tissues. Although the exact reasons for these problems were never fully identified, in the later production phase (i.e. the majority of pools), the levels of *E. coli* and mitochondrial DNA stabilized at 2.9% and 1.8%, respectively. Special care was needed to remove vector sequence from the Fosmid vector/insert junction. All contig ends mapped with BLAST to the vector sequence were therefore removed at the length of 30 bp. Finally, we also removed all contigs shorter than 500 bp because their detailed investigation usually revealed severe mis-assembly problems, such as composition of only unpaired (orphan) reads, wrong paired end orientation, abundance of abnormal insert sizes between paired reads etc.

After contamination screening, we optimized assembly performance by varying the word length *k* for the CLC bio *de novo* assembly algorithm in the CLC Assembly Cell software. Results of assembling FPs with different values of *k* (*k =* 21…57) were compared by means of FRC curves [11] (Additional file 1: Figure S3), and we concluded that the optimal value of *k* for these assemblies was 51. We also tried varying parameters related to De Bruijn-graph “bubbles”, which are typically used to allow for the assembly of heterozygous alleles. As the FPs should be essentially haploid, this parameter was not expected to affect the assembly performance, and indeed we observed no differences when varying the parameters for bubble length, bubble fraction, and bubble length difference (data not shown).

### Resolving repeats in the assembly

**Figure 3:**
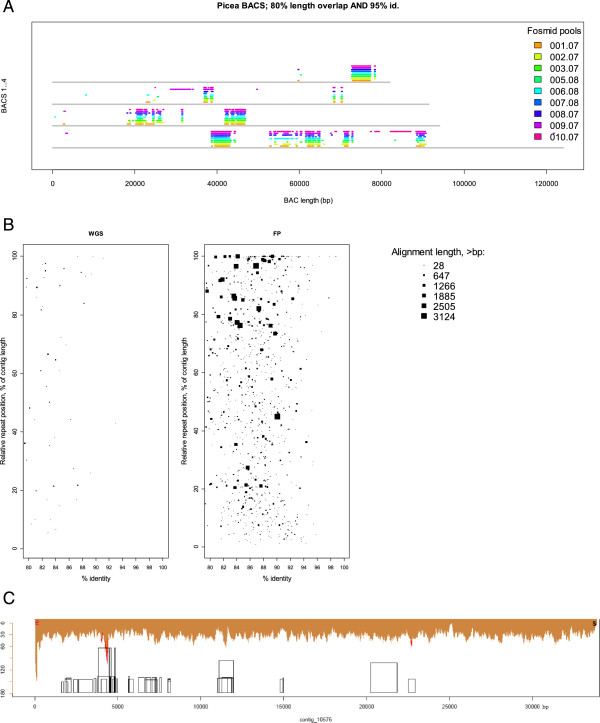
**Repeat positions in the assembly. A**. Four available *Picea abies* BAC sequences. Contigs assembled within each of the nine test pools were mapped to the 4 BACs (horizontal grey lines) available for the *P. abies* genome. Multiple hits to the same BAC regions from different FPs were identified (color bars). Multiple hits from the same FP were also common, although they are not shown here because of a lack of resolution. **B**. Library repeats mapped to contigs assembled from two different sequencing pipelines: whole genome sequencing versus FP sequencing. From both WGS and FP assemblies (available from the database of genome assembly lock v. 1.0), random subsets of 1000 contigs (min length 5000 bp) were aligned against the repeat library presented for spruce genome in [7]. Horizontal coordinates reflect percent identity of contig BLAST hits against sequences from the repeat library. Vertical axes represent the total length of each contig, so that hit points are rescaled and placed proportionally to the length of the respective contig. Filled boxes represent length of a contig hit to a repeat sequence, at the appropriate position relative to the contig length. The hit lengths range from 18 bp (invisible) to over 3000 bp. **C**. Example contig likely assembled to full original length, with read coverage, vector fragments, and repeat regions. Horizontal axis: length of the contig in base pairs. Vertical axis: read coverage; brown color: unique read mapping; red color: redundant read mapping. Letters S and E: 3′ and 5′ termini of the vector sequence, respectively (~40 bp long). Boxes: repeat regions in the contig. Height denotes original length in the repeat library, width represents locally aligned region of the repeat. **A**, **B**, **C**.

High copy-number repeats make up about 70% of the Norway spruce genome [7]. Because of this, one of the main advantages of using the FP technology in this project was that it gave us the ability to resolve repeat regions more efficiently. In the production mode, we observed that most of the pools yielded >20 Mbp, more than half of the length expected, assembled in contigs greater than 4,000 bp in length(Figure 2A). Most of the pools had at least 95% of the contigs covered by paired reads to a depth of greater than 10X (Figure 2C). Figure 3A demonstrates how the same genomic regions of four previously sequenced BACs [7] mapped to numerous contigs from different FPs. However, sequence identity between different instances of the same repeat was not perfect. We hoped that it would be possible to resolve a major proportion of repeats within an FP, and that this proportion would be higher than when using the WGS approach. Indeed, mapping known *P. abies* repeats to WGS- and FP-derived contigs showed a dramatic increase in the number of resolved repeats observed in the FP assemblies compared to the WGS assembly (Figure 3B). In WGS, very few repeat-like regions were found in the middle of contigs and they were usually short. In contrast, FP contigs were rich in repeats, and in many cases hits (filled boxes in Figure 3B) matched a significant fraction of the corresponding library sequence (up to 100%). BLAST alignments confirmed that the repeat regions typically had less than perfect identity to the library sequences (80 - 99% homology) and hence to each other, thus repeats within a FP could often be resolved unambiguously (Figure 3C).

We summarized the repeat situation in the FP assemblies used for genome assembly lock v. 1.0 as follows: there were 457 FPs with around 365,000 individual fosmid inserts sampled. They were assembled to 13,090,107 contigs (including short ones which were later excluded from the assembly), i.e. 28,643 contigs per pool instead of expected 600-1000 full fosmid inserts. Mapping the contigs to the repeat library gave 40,559,402 BLAST hits with identity higher than 78%. Thus an average contig sequence contained 3.1 identified repeat regions against potentially versus 2 potential repeats at its flanks that we failed to assemble. On the other hand, 55.4% of the contigs did not contain any repeat regions identifiable with this method. The assembly termination in such contigs could be explained by either unknown repeats or other reasons. Nonetheless, more than 60% of identifiable repeats were resolved. For comparison, in the four BACs we identified 1853 repeat hits which gives a very close estimate of repeat content per sequence length (one repeat per 200…250 bp).

### Sampling on a whole-genome scale

**Figure 4:**
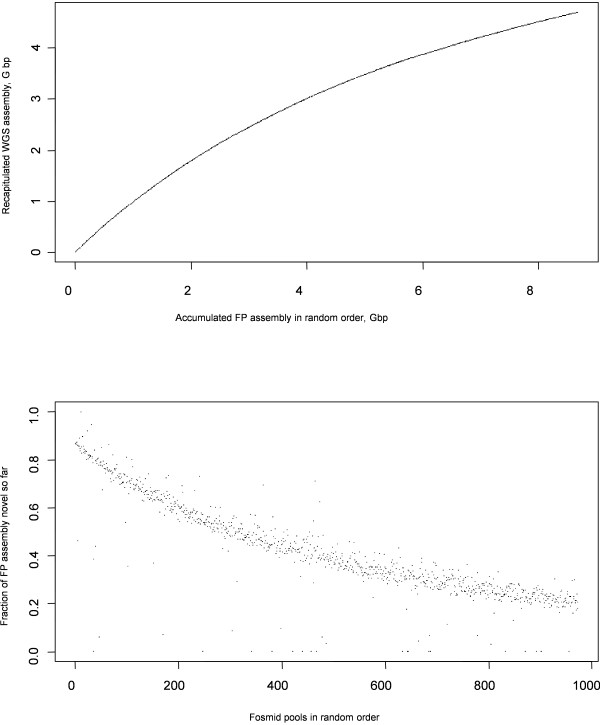
**Discovery of novel regions of the genome by recurrent sampling with Fosmid pools.** The diploid whole genome shotgun (WGS) assembly was used as a reference. All the available 972 FPs assemblies were aligned against the WGS assembly. Only contigs longer than 1000 bp and alignments longer than 500 bp (percent identity >99%) were counted. BLAST mapping masked WGS sequences at discrete points every 500 bp along the contig coordinates. Cumulative statistics were collected in random FP order. Upper panel: Total assembled length of FP (horizontal axis) versus the summary length of WGS contigs having matches to FP contigs (vertical axis). Lower panel: Fraction of current FP contig length mapped to WGS regions which had not been found (i.e. not masked) in previous FP assemblies.

By aligning the FP contigs to the WGS contigs, we were able to demonstrate that the pools sample the known genome sequence evenly. At the point in production where the whole *P. abies* genome had been sampled around two-fold with Fosmids, we utilized the available FP assemblies and the WGS assembly to model this process. We randomly ordered FP contig sets, mapped them consecutively to the WGS assembly, and recorded how much sequence from the latter was brought in for the first time by a given FP. The cumulative plot (Figure 4) demonstrated that, at each step, novel genome fragments were gained. In other words, there was no significant bias towards sampling particular genomic regions (Figure 4), and we approached the full genome complement asymptotically.

### Scaffolding with WGS data

Since mate-pair sequencing libraries are costly and time-consuming to generate, it is generally impracticable to generate data for each FP using this approach. Instead, in this project we explored the possibility of scaffolding each pool using WGS data. Two WGS libraries of different insert sizes (paired end 625 bp and jumping 2.4 Kbp) were generated from the diploid tissue and sequenced to depths of 20X and 25X physical coverage, respectively. For each FP, the two libraries were mapped to the pool contigs and used for scaffolding with a new software tool, BESST [12], available at https://github.com/ksahlin/BESST. Special care was taken to avoid false links caused by reads from another part of the genome being mapped to a pool, firstly by imposing very strict mapping criteria (a maximum of 1 mismatch and no indels per read), and secondly by disqualifying all potential joins with any ambiguities in the linking graph. As exemplified by the test pools, this scaffolding procedure increased the total length of contigs longer than 10 Kbp from 43% to over 75% of the expected assembly size (Figure 1).

## Discussion and conclusion

We developed a procedure for FP preparation, sequencing, assembly, quality control, and contig post-processing. We then applied the technology in a hitherto unparalleled large-genome sequencing project. The repeat-rich 20 Gbp Norway spruce genome was decoded using solely massive parallel sequencing [7]. The FP pipeline became an indispensable part of the project. We found, during the early stages of the work, that it was possible to identify and characterize biologically relevant functional and structural elements of the genome, such as the different types of repeat and regions homologous to known plant genes, and to assess the similarity of the results to those of WGS assembly (Additional file 1: Figure S4). We also demonstrated and utilized the ability of FPs to assemble longer contigs and to incorporate repeats at much higher rates compared to WGS. The entire FP processing pipeline could be run on parallel nodes with modest memory capacity (less than 8 GB) and each pool required less than 6 CPU hours; the assembly part usually took less than 0.5 hours. For comparison, the diploid WGS assembly was run on a 40 CPU computer with 1 TB RAM for more than 100 wall clock hours.

How scalable is FP sampling of this large genome, which should by definition be random? Even after having sampled around 40 Gbp from the genome with FPs, approximately 20% of each new FP appeared to contribute novel sequence. It is often important to estimate the distribution of gaps in a WGS assembly. To do this, one can map Fosmid contigs over the WGS set. This estimation is likely to be biased by a non-uniform distribution of FP contig length. Because of this, only contigs within a certain length range (e.g. 20-40 Kbp) should be used, although it will still not be possible to estimate gaps longer than 20 Kbp.

However, the major challenge in using FPs is the merging of FP assemblies into the WGS assembly. State-of-the-art scaffolders are unable to deal with two alternative contig sets. Furthermore, the WGS contigs are by definition unique, whereas FP contig sets may overlap between pools. In diploid assemblies, certain pairs of homologous WGS contigs can represent two alternative allelic forms of the same region of the genome, while FP contigs should effectively lack such redundancy. We addressed this problem using a newly developed software package, GAM-NGS [13], which finds matching (aligned) regions between FP and WGS assemblies by ‘by taking into account both these constraints and read mapping information.

## Electronic supplementary material

Additional file 1: Table S1: Read QC parameters of fastq_quality_filter (FASTX toolkit v. 0.0.13). **Table S2**: Expected vs. evaluated number of fosmids per pool. **Figure S1**: Total length of contigs per pool as function of coverage. **Figure S2**: Feature response curves used for optimization of read quality filtering before assembly. **Figure S3**: Feature response curves used for optimization of *k*-mer length for CLC assembly. **Figure S4**: Mapping of WGS to FP contigs. (DOCX 601 KB)
