# Supplementary material for: Efficient de novo assembly of large and complex genomes by massively parallel sequencing of Fosmid pools
Source: BMC Genomics. 2014 Jun 6;15(1):439. doi: 10.1186/1471-2164-15-439 (PMC4070561; doi:10.1186/1471-2164-15-439)
Supplement: Supplementary file 1 — Additional file 1: Table S1: Read QC parameters of fastq_quality_filter (FASTX toolkit v. 0.0.13). Table S2: Expected vs. evaluated number of fosmids per pool. Figure S1: Total length of contigs per pool as function of coverage. Figure S2: Feature response curves used for optimization of read quality filtering before assembly. Figure S3: Feature response curves used for optimization of k-mer length for CLC assembly. Figure S4: Mapping of WGS to FP contigs. (DOCX 601 KB) [file 12864_2013_6124_MOESM1_ESM.docx]

## Additional file 1: Supplementary material

Supplementary table 1. Read QC parameters of fastq_quality_filter (FASTX toolkit v. 0.0.13).

| **Strictness** | **-q: minimum quality score to keep** | **-p: minimum percent of bases that must have [-q] quality** |
| --- | --- | --- |
| High (b) | 20 | 100 |
| Medium (a) | 20 | 95 |
| Low (c) | 20 | 85 |

Supplementary table 2. Expected vs. evaluated number of fosmids per pool.

| **Pool** | **Expected** | **Actual** |
| --- | --- | --- |
| 384_rerun | 384 | 301 |
| 768_rerun | 768 | 669 |
| 001 | 1000 | 594.5 |
| 002 | 1000 | 694 |
| 003 | 1000 | 884 |
| 005 | 1000 | 913.5 |
| 006 | 1000 | 615.5 |
| 007 | 1000 | 963 |
| 008 | 1000 | 552 |
| 009 | 1000 | 657 |
| 010 | 1000 | 534 |
| Luci106 | 106 | 108 |
| Luci516 | 516 | 500 |
| Luci1000 | 1000 | 1091 |
| Luci2500 | 2500 | 2592 |
| Luci5000 | 5000 | 4334 |

Supplementary figure 1. Total length of contigs per pool as function of coverage.

Reduced levels of coverage were simulated by random sub-sampling of reads as 25x, 50x, and 75x in regard of the sampled genome size (40 Kbp X expected no. of fosmids).


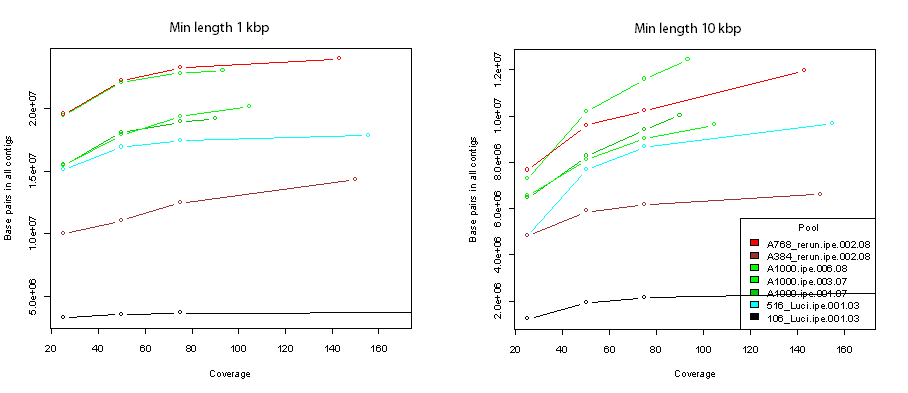


Supplementary figure 2. Feature response curves used for optimization of read quality filtering before assembly.

Values given in Suppl. Table 1 were chosen after testing their impact on the CLCbio assembly.

Tests on five example fosmid pool contig sets (1B2, 1C1, 1E1, 1G8, 1G12) are shown.

9 different assembly features versus assembled length indicated potential false positive rate:

LOW_COV_PE, low read coverage areas (all aligned reads).

HIGH_COV_PE, high read coverage areas (all aligned reads).

LOW_NORM_COV_PE, low paired-read coverage areas (only properly aligned pairs).

HIGH_NORM_COV_PE, high paired-read coverage areas (only properly aligned pairs).

COMPR_PE, low CE-statistics computed on PE-reads.

STRECH_PE, high CE-statistics computed on PE-reads.

HIGH_SINGLE_PE, high number of PE reads with unmapped pair.

HIGH_SPAN_PE, high number of paired reads with the other read mapped to a different contig.

HIGH_OUTIE_PE, number of mis-oriented or too distant PE reads higher than a threshold.

COMPR_MP, low CE-statistics computed on MP reads.

STRECH_MP, high CE-statistics computed on MP reads.

HIGH_SINGLE_MP, high number of MP reads with unmapped pair.

HIGH_SPAN_MP, high number of MP reads with pair mapped in a different contig/scaffold.

HIGH_OUTIE_MP, high number of mis-oriented or too distant MP reads}


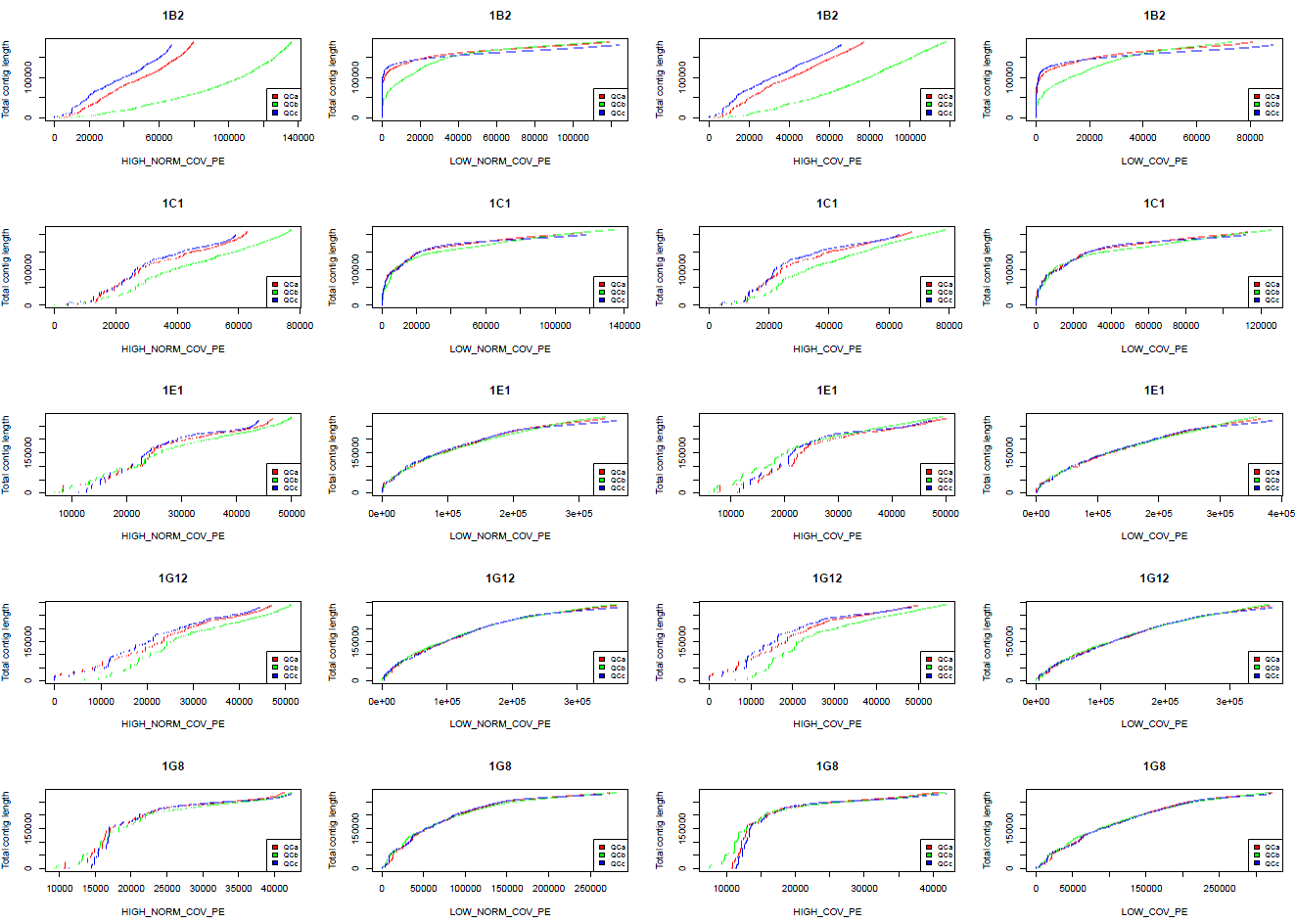


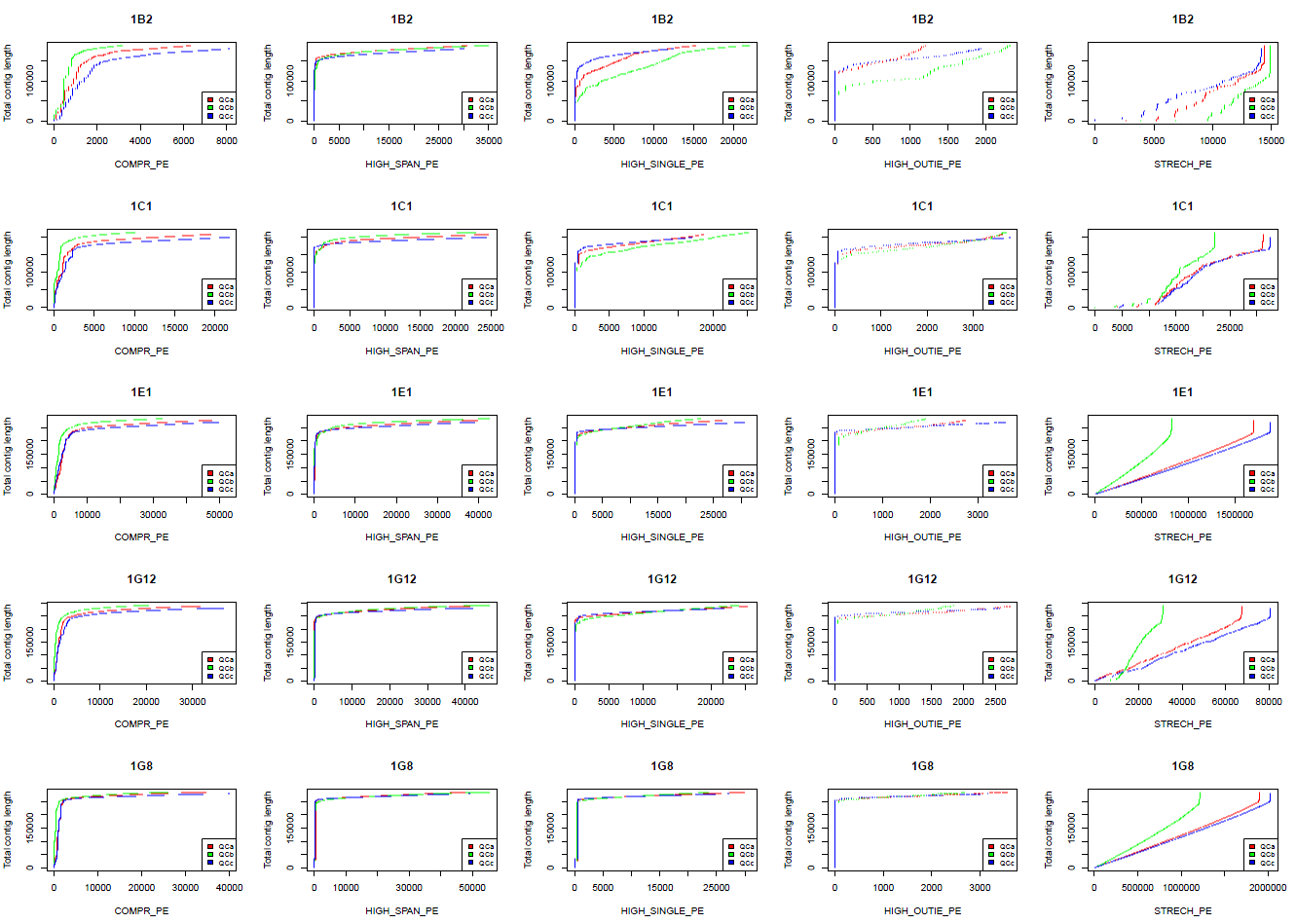


Supplementary figure 3. Feature response curves used for optimization of *k*-mer length for CLC assembly.

Values of k={27,35,43,51} bp were tested as parameters to CLC de novo assembler.

Tests on three example fosmid pool contig sets (1E1, 1G8, 1G12) are shown.

Feature legend is the same as in Suppl. Fig 2.


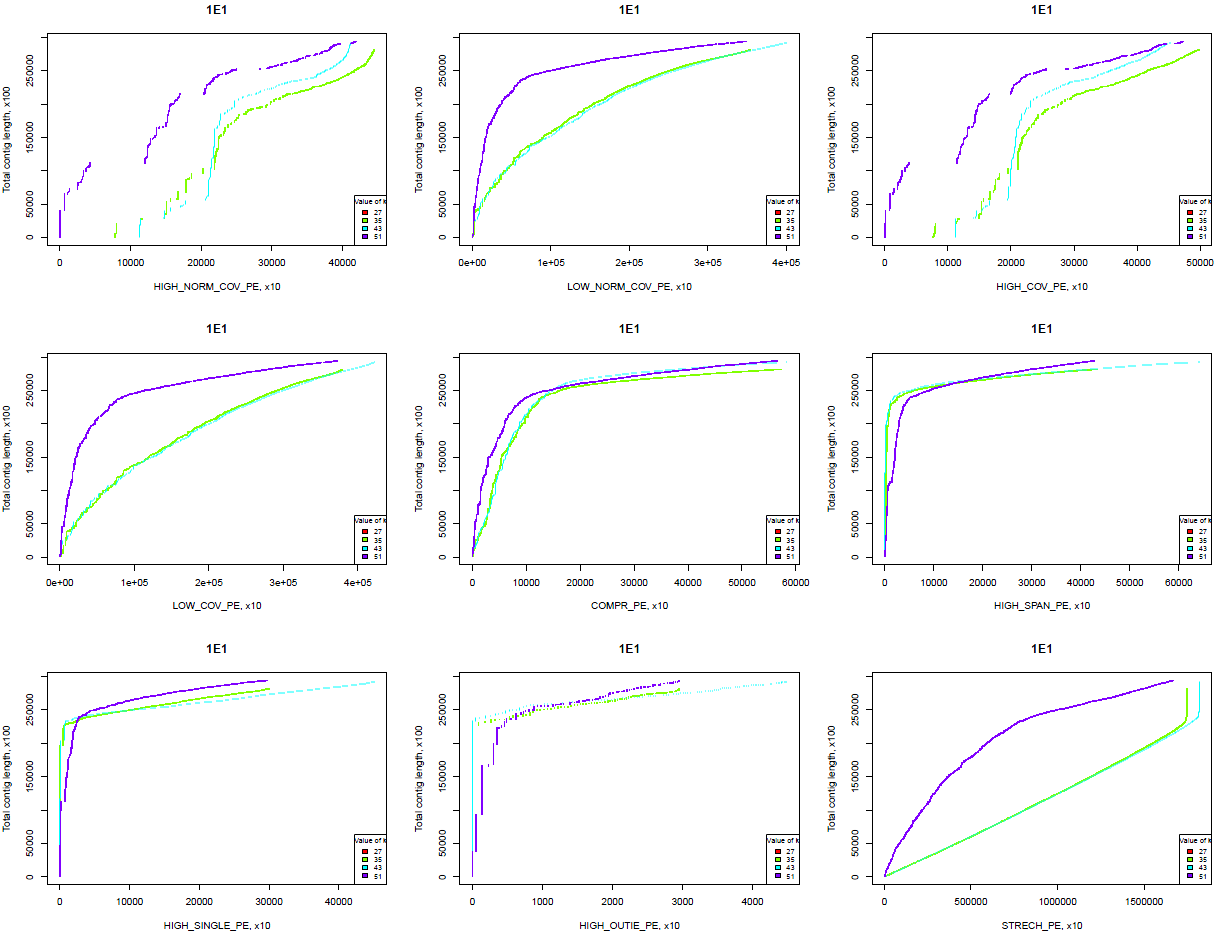


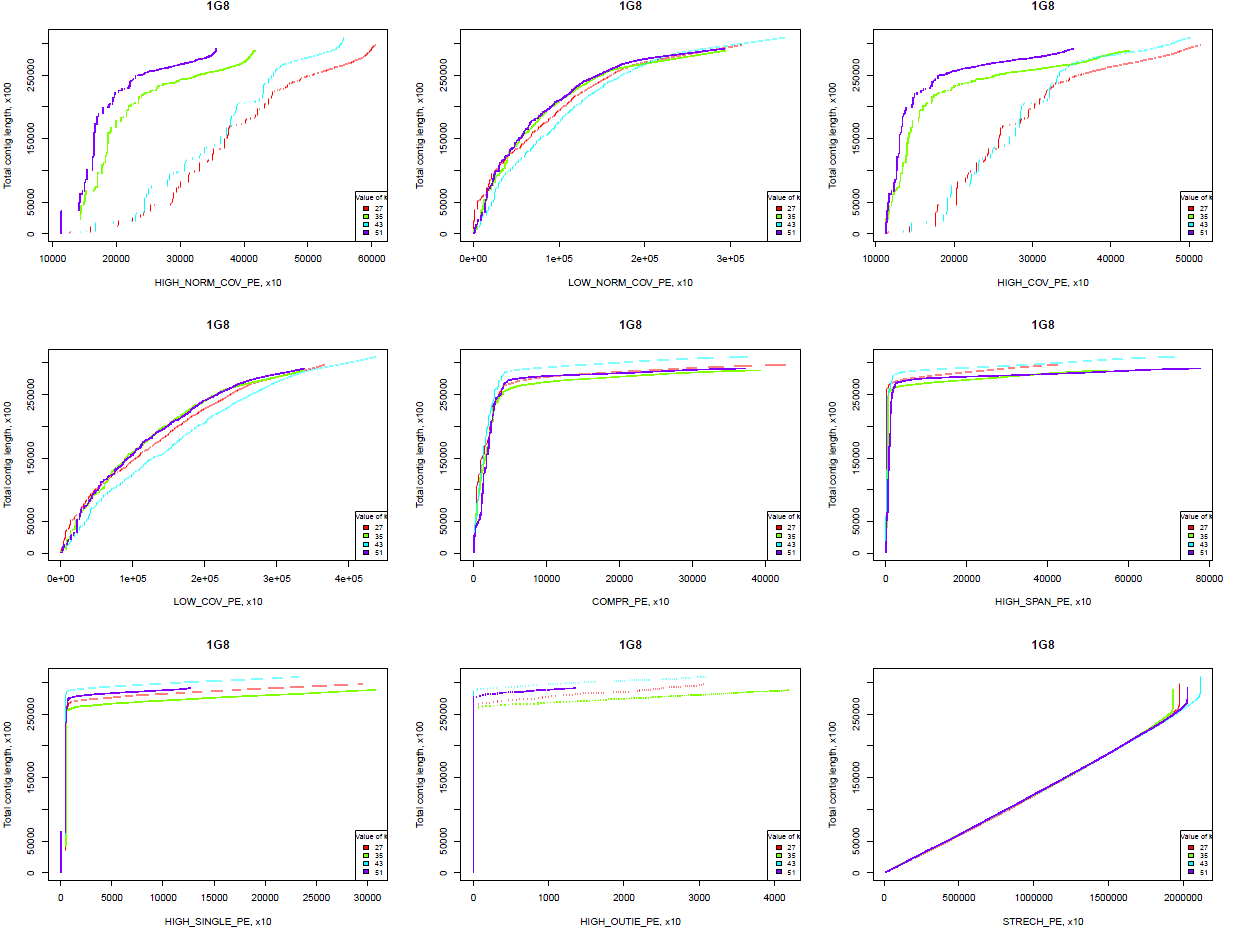


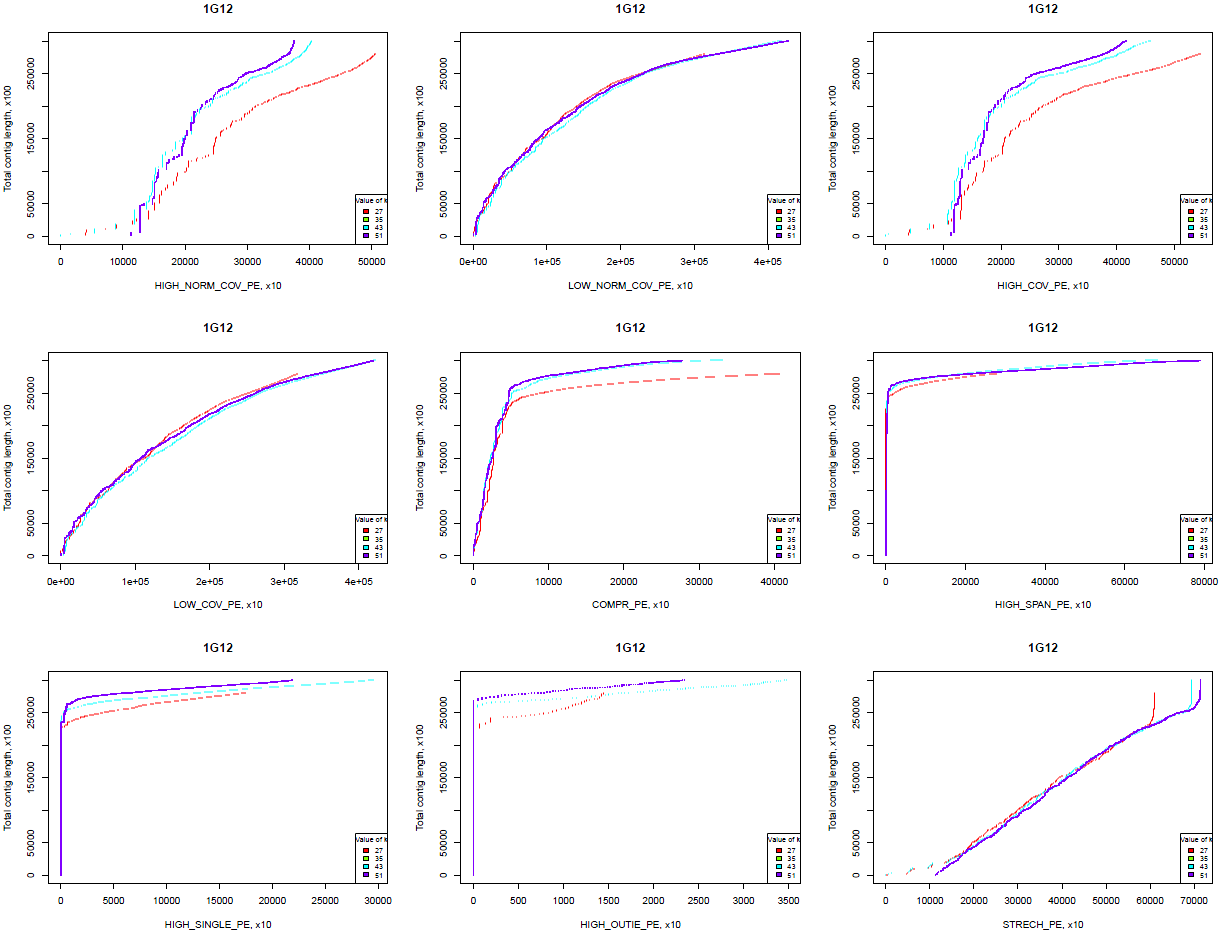


Supplementary figure 4. Mapping of WGS to FP contigs.

30 example FP contigs that had been assembled to the expected length (~40 Kbp) were aligned with BLAST against the diploid whole genome shotguns assembly. Longer red stretches likely indicate different allele versions present as distinct WGS contigs.
